# Supplementary material for: Retinal Microcirculation Changes in Crohn’s Disease Patients under Biologics, a Potential Biomarker of Severity: A Pilot Study
Source: J Pers Med. 2022 Feb 7;12(2):230. doi: 10.3390/jpm12020230 (PMC8878992; doi:10.3390/jpm12020230)
Supplement: Supplementary file 1 [file jpm-12-00230-s001.zip › jpm-1545288-supplementary.pdf]

Online-Only Supplements

Table S1. Age- and sex-adjusted analysis of covariance: association of the main microvascular variables according to clinical activity (n=74).

	Clinical activity		
	**Yes**, N = 21	**No**, N = 53	**p-value**[1]
**FAZ area** (mm^2)	0.29 (0.02)	0.26 (0.01)	0.14
**Whole SCP-VD** (%)	47.8 (0.8)	47.7 (0.5)	0.78
**Parafoveal SCP-VD** (%)	50.8 (0.9)	50.4 (0.5)	0.55
**RPC-VD** (%)	52.3 (0.6)	51.1 (0.38)	0.10
**ln (SCP-FI)** (mm^2)	0.30 (0.02)	0.30 (0.01)	0.88
**ln (DCP-FI)** (mm^2)	-0.79 (0.13)	-1.03 (0.08)	0.07
**CCP-FI** (mm^2)	2.10 (0.03)	2.12 (0.02)	0.60

Data are mean (SEM).
[1]Model adjusted for sex and age: all potential confounders had a p-value ≥0.20 on univariate analysis

<u>Abbreviations:</u> CCP, choriocapillaris plexus; DCP, deep capillary plexus; FAZ, foveal avascular zone; FI, flow index; SCP, superficial capillary plexus; RPC, radial peripapillary capillaries; SEM, standard error of the mean; VD, vascular density

Table S2. Age-, sex- and tobacco use- adjusted analysis of covariance: association of the main microvascular variables with deep activity status (n=68).

	Deep activity		
	Yes, N = 34	**No**, N = 34	**p-value**[1]
**FAZ area** (mm^2)	0.29 (0.02)	0.26 (0.02)	0.31
**Whole SCP-VD** (%)	47.4 (0.6)	48.0 (0.7)	0.32
**Parafoveal SCP-VD** (%)	50.2 (0.7)	50.8 (0.8)	0.29
**RPC-VD** (%)	51.6 (0.5)	51.6 (0.5)	0.99
**Ln (SCP-FI)** (mm^2)	0.29 (0.02)	0.32 (0.02)	0.26
**Ln (DCP-FI)** (mm^2)	-0.85 (0.1)	-1.0 (0.1)	0.17
**CCP-FI** (mm^2)	2.12 (0.02)	2.14 (0.02)	0.80

Data are mean (SEM).
[1]Model adjusted for sex, age and tobacco: tobacco was the only potential confounder with a p value < 0.20 (Chi 2 test, p=0.18)

<u>Abbreviations:</u> CCP, choriocapillaris plexus; DCP, deep capillary plexus; FAZ, foveal avascular zone; FI, flow index; SCP, superficial capillary plexus; RPC, radial peripapillary capillaries; SEM, standard error of the mean; VD, vascular density
